# Menopausal symptoms and risk of coronary heart disease in middle-aged women: A nationwide population-based cohort study

**DOI:** 10.1371/journal.pone.0206036

**Published:** 2018-10-18

**Authors:** Ching-Hui Huang, Cheng-Liang Li, Chew-Teng Kor, Chia-Chu Chang

**Affiliations:** 1 Division of Cardiology, Department of Internal Medicine, Changhua Christian Hospital, Changhua, Taiwan; 2 Department of Beauty Science and Graduate Institute of Beauty Science Technology, Chienkuo Technology University, Changhua, Taiwan; 3 School of Medicine, College of Medicine, Kaohsiung Medical University, Kaohsiung, Taiwan; 4 Medical Research Center, Department of Internal Medicine, Changhua Christian Hospital, Changhua, Taiwan; 5 Department of Internal Medicine, Kuang Tien General Hospital, Taichung, Taiwan; 6 Department of Nutrition, Hungkuang University, Taichung, Taiwan; Erasmus MC, NETHERLANDS

## Abstract

**Objective:**

To assess the relationship between coronary heart disease (CHD) and menopausal symptoms in middle-aged women in Taiwan.

**Patients and methods:**

The present study identified 14,340 symptomatic menopausal women without a history of CHD from the Taiwan National Health Insurance Research Database from January 1, 2000, to December 31, 2013. A total of 14,340 age- and Charlson-comorbidity-index-score-matched asymptomatic women were used as controls. Possible comorbidity-attributable risks of CHD were surveyed to assess whether the symptomatic menopausal cohort had a higher incidence of CHD.

**Results:**

The incidence of CHD was higher in the symptomatic menopausal cohort than in the control cohort (17.18 vs. 12.05 per 1000 person-years). After adjustment in multivariate Cox analysis, the risk of CHD was significantly higher in the symptomatic menopausal cohort (adjusted hazard ratio = 1.344, 95% confidence interval [CI] = 1.262–1.43, *P* < 0.001) than in the control cohort. In the symptomatic menopausal cohort, the risk of CHD was significantly higher in all subgroups, except for the hormone therapy (HT) subgroup. Patients undergoing HT had a nonsignificantly higher risk of CHD, regardless of the presence or absence of menopausal symptoms.

**Conclusion:**

This large-scale longitudinal retrospective cohort study revealed that menopausal symptoms are an independent risk factor for CHD. Moreover, our findings indicate that HT has a nonsignificant effect on the risk of CHD.

## Introduction

The incidence of cardiovascular diseases (CVDs) has increased in postmenopausal women [1,2]. The prevalence of hot flashes and other menopausal symptoms has been reported to be up to 80% in menopausal women, and the prevalence is influenced by various factors, such as age, ethnicity, education, smoking, and mood [3,4]. Increasing bodies of evidence indicate that menopausal symptoms are associated with risk factors for coronary heart disease (CHD) and surrogate markers for CHD and clinical CVD [5–7]. Middle-aged women with menopausal symptoms exhibit adverse changes in CVD risk factors and, consequently, increased CVD risk [8]. However, it remains unclear whether the presence of menopausal symptoms can truly predict clinical CVD events or whether CVD is primarily explained by associated CVD risk factors. To date, no consensus has been reached on this topic.

The present study investigated whether menopausal symptoms predict the risk of CHD in a cohort of women with medical comorbidity-attributable risks.

## Materials and methods

### Data source

All data on demographics, clinic visits, hospital admissions, prescription records, and disease status were retrieved from the Taiwan National Health Insurance Research Database (NHIRD). The NHIRD contains the claims data of all insurants of Taiwan’s National Health Insurance (NHI) program, which provides coverage to 99% of the population of Taiwan. The diagnostic codes for identifying diseases in the NHIRD are based on the International Classification of Diseases, Ninth Revision, Clinical Modification (ICD-9-CM). All personal identifiers were encrypted by the National Health Insurance Administration prior to study commencement. This study was exempt from full review and was approved by the Institutional Review Board of Changhua Christian Hospital (Approval Number 170411). The requirement for informed consent was waived.

### Patients

**Fig 1:**
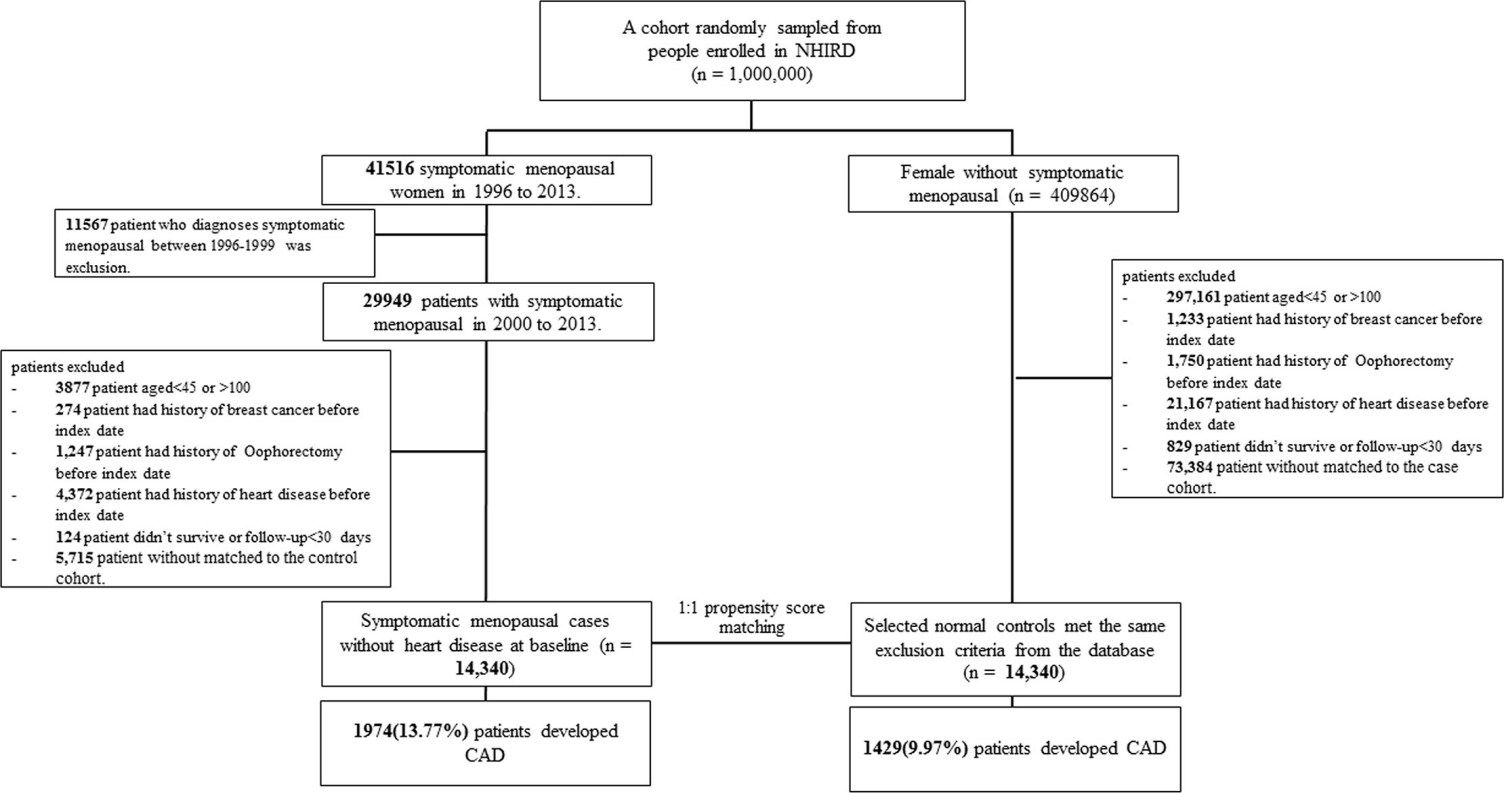
Flowchart of selection of patients with menopausal symptoms from the NHIRD and selection of controls from age-, CCI-score-, index-date-, and propensity-score-matched asymptomatic women.

A cohort of approximately 1,000,000 patients over the period from 1996 to 2013 was randomly sampled from the NHIRD. A total of 41,516 women with menopausal symptoms were selected (ICD-9-CM 627.2). All diagnoses of symptomatic menopause were made by a gynecologist. We excluded 11,567 women with menopausal symptoms who received a diagnosis between 1996 and 1999 because the demographic data collected during these years were incomplete or had missing records. The index date (enrollment date) was defined as the first date when an eligible woman was diagnosed with symptomatic menopause by using the diagnostic code ICD-9-CM 627.2. Patients with CHD, heart failure, breast cancer, or oophorectomy before the index date; those aged <40 or >100 years; those with incomplete demographic data; and those who did not survive for >30 days after receiving a diagnosis of symptomatic menopause were excluded. The control cohort comprised patients without a history of CHD, heart failure, breast cancer, oophorectomy, or menopausal symptoms before the index date. The control cohort was selected through 1:1 propensity score matching. A flowchart of the cohort selection process is presented in **Fig 1**. To balance the measured covariates between the two study cohorts, we calculated the propensity score by using multivariate logistic regression to predict the likelihood of menopausal symptoms for each patient. For each patient with menopausal symptoms, one control without menopausal symptoms was selected through matching by age, calendar year of index date, and propensity score. We used the nearest-neighbor algorithm with a caliper of 0.1 SD units to generate matched pairs. The details of the propensity score model are described in **S1 Table.**

### Validation of symptomatic menopause diagnosis

For diagnostic validity, the diagnosis of symptomatic menopause (ICD-9-CM 627.2) had to be made at least three times by gynecologists. To increase the reliability of the diagnosis, we conducted sensitivity tests of the diagnostic code combined with corresponding medications. Symptomatic menopause manifests through various somatic symptoms, including vasomotor symptoms (i.e., hot flushes and night sweats), urogenital symptoms, headaches, and joint pain. In addition, symptomatic menopausal transition includes psychiatric symptoms, such as depressive and anxious mood, sleep disturbance, and feelings of panic [9]. The corresponding medications included antidepressants, antiepileptics (gabapentin), hypotensive agents (clonidine), osteoporosis medications (raloxifene), beta-blocking agents, hormone therapy (HT), antipsychotics, anxiolytics, hypnotics, and sedatives. If any one of these medications were prescribed after diagnostic code allocation, we considered the diagnostic code to be accurate. The diagnostic accuracy rate was 91.71% (**S2 Table**).

### Outcome measures and relevant variables

We examined ICD-9-CM codes in the patients’ records to determine outcomes and comorbidities. Both the symptomatic menopausal and control cohorts were followed up from the date of study enrollment to the date of the first occurrence of CHD, date of withdrawal from the NHI program, or end of 2013. The date of withdrawal from the NHI program has been recognized as an accurate and reliable proxy for the date of death in Taiwan [10–12]. CHD was identified on the basis of the corresponding diagnostic codes (ICD-9-CM 410–414) recorded for at least three outpatient clinic visits or at least one hospital admission. All data on comorbidities, including hypertension, hyperlipidemia, diabetes mellitus, chronic kidney disease, stroke, chronic obstructive pulmonary disease, peripheral artery occlusive disease, and dysrhythmia, were collected from the NHIRD. Charlson comorbidity index (CCI) scores were used to quantify baseline comorbidities. The CCI is the most extensively applied comorbidity index for predicting mortality [13,14]. It identifies the comorbidities of patients and weights them based on the adjusted risk of mortality or health resource use; the sum of all the weights then provides a single comorbidity score. A score of 0 indicates that no comorbidities were present in patients. A higher score indicates that the predicted outcome will more likely result in a higher risk of mortality or higher health resource use. Additionally, long-term medication use is believed to be associated with CHD outcomes in menopausal women, and in this study, the long-term use of medications, namely antidiabetic agents, statins, antihypertension medications (e.g., angiotensin-converting enzyme inhibitors or angiotensin II receptor blockers), calcium channel blockers, betablockers, diuretics, and HT, was recorded according to the Anatomical Therapeutic Chemical codes defined by the World Health Organization. Patients who used these medications for more than 90 consecutive days prior to receiving a diagnosis of CHD were defined as medication users. If the medications were stopped for more than 180 days during the follow-up period, the corresponding data were censored.

### Individual socioeconomic status

This study used the monthly income calculated from the NHI premium as a proxy for individual socioeconomic status (SES). Individuals were classified into three income groups: (1) low (<NT$15,840 per month); (2) medium (NT$15,840–NT$25,000 per month); and (3) high (>NT$25,000 per month). The classification was based on the minimum monthly wage for the year 2006 in Taiwan (NT$15,840). According to data released in 2015 by the Ministry of Labor in Taiwan, the average salary after graduation was NT$25,000.

### Statistical analysis

The demographic data and other clinically relevant data of both cohorts are presented as proportions and means ± SD. A standardized difference (StD) of more than 0.1 indicated significant heterogeneity between the two cohorts. The Cox proportional hazard model was used to estimate the relative risk of CHD in the symptomatic menopausal and control cohorts. Adjusted hazard ratios (aHRs) were estimated using multivariate Cox analysis, which was adjusted for confounders, namely age, CCI score, monthly income, comorbidities, and long-term medication use. Because comorbidities may not only be present at baseline but also develop during follow-up, comorbidities were modeled using nonreversible time-dependent binary covariates for event analyses. A competing risk is an event that either hinders the development of the event of interest or modifies the chance of the occurrence of this event. Because CHD risks might compete with the risk of death, the Cox proportional hazard model with competing risks (Fine–Gray model) of death was used to estimate the relative risk of CHD in the symptomatic menopausal cohort compared with the nonsymptomatic menopausal cohort. This model is a proportional hazards model employed for subdistribution analysis [15]. In this study, a test of interaction was conducted to determine observable subgroup effects. For example, to test the interaction between symptomatic menopause and comorbidity, the interaction in the Cox proportional hazard model was set as follows: λ(t) = λ_0_(t)*exp*(*MS* + comorbidity + *MS* × comorbidity disease + ⋯), where MS is symptomatic menopause and comorbidity is a binary variable, which is calculated using the following formula: $comorbidity=\left\{ \begin{array}{cc} 1 & at\;least\;1\;comorbidity\\ 0 & none \end{array}\right.$.

Patients’ histories of HT and CHD were also analyzed using the multivariate Cox model. All statistical analyses were performed using SAS 9.4 software (SAS Institute Inc., Cary, NC, USA). Two-tailed *P* values of <0.05 were considered statistically significant.

## Results

### Baseline characteristics of women according to menopausal symptoms

**Table 1** lists the baseline demographic data of the enrolled patients. The mean age of the patients in the symptomatic menopausal cohort was 52.2 ± 6.6 years. At baseline, 1993 (13.9%), 992 (6.92%), 683 (4.76%), 173 (1.21%), 210 (1.46%), and 55 (0.38%) patients had hypertension, hyperlipidemia, diabetes mellitus, chronic kidney disease, a history of stroke, and peripheral arterial occlusive disease, respectively. Furthermore, 8.88%, 2.8%, and 2.33% of the patients in the symptomatic menopausal cohort received antihypertensive medication, antidiabetic agents, and statins, respectively. The CCI scores were balanced between the cohorts, and an StD of >0.1 was not observed between the symptomatic menopausal and control cohorts in terms of comorbidities and medication use, except for HT. The proportion of women who received HT was higher in the symptomatic menopausal cohort than in the control cohort (20.68% vs. 1.97%; StD = 0.618). The mean duration of HT was higher in the symptomatic menopausal cohort than in the control cohort (3.3 ± 3.4 years vs. 1.3 ± 2.3 years; StD = 0.689). The present study also analyzed the patients’ income status. In the symptomatic menopausal cohort, 6327 (44.12%), 5077 (35.4%), and 2936 (20.47%) patients had monthly incomes of <NT$15,840, NT$15,840–NT$25,000, and >NT$25,000, respectively. Moreover, a StD of >0.1 was not observed between the symptomatic menopausal and control cohorts across the three income categories.

**Table 1:** Baseline characteristics of symptomatic menopausal and control cohorts.

	No Symptoms	With Symptoms	total	StD^a^
Sample size	14,340	14,340	28,680	
Age	52.2±6.71	52.22±6.59	52.21±6.65	0.003
Charlson’s comorbidity index score				
0	8050(56.14%)	8050(56.14%)	16100(56.14%)	0.000
1–2	5425(37.83%)	5425(37.83%)	10850(37.83%)	0.000
≥3	865(6.03%)	865(6.03%)	1730(6.03%)	0.000
Monthly income, NTD				
<15840	6200(43.24%)	6327(44.12%)	12527(43.68%)	0.018
15840–25000	5254(36.64%)	5077(35.4%)	10331(36.02%)	0.026
≥25000	2886(20.13%)	2936(20.47%)	5822(20.3%)	0.009
Comorbidities at baseline				
Hypertension	1856(12.94%)	1993(13.9%)	3849(13.42%)	0.028
Hyperlipidemia	896(6.25%)	992(6.92%)	1888(6.58%)	0.027
Diabetes mellitus	869(6.06%)	683(4.76%)	1552(5.41%)	0.057
Obesity	56(0.39%)	55(0.38%)	111(0.39%)	0.001
CKD	198(1.38%)	173(1.21%)	371(1.29%)	0.015
Stroke	182(1.27%)	210(1.46%)	392(1.37%)	0.017
COPD	732(5.1%)	659(4.6%)	1391(4.85%)	0.024
PAOD	32(0.22%)	55(0.38%)	87(0.3%)	0.029
Dysarrhythmia	134(0.93%)	175(1.22%)	309(1.08%)	0.028
Long-term use of medications^b^				
Anti-hypertension	1188(8.28%)	1274(8.88%)	2462(8.58%)	0.021
Diabetic drugs	498(3.47%)	401(2.8%)	899(3.13%)	0.039
Statin	280(1.95%)	334(2.33%)	614(2.14%)	0.026
Hormone therapy (HT)	283(1.97%)	2966(20.68%)	3249(11.33%)	0.618
Estrogens	182(1.27%)	1405(9.8%)	1587(5.53%)	0.380
Progestogens	140(0.98%)	1001(6.98%)	1141(3.98%)	0.311
Progestogens and estrogens in combination	92(0.64%)	1479(10.31%)	1571(5.48%)	0.435
HT duration (years)	1.3±2.3	3.3±3.4	3.0±3.3	0.689
Propensity score	0.22±0.04	0.22±0.04	0.22±0.04	0.003

^a^StD, standardized difference of more than 0.1 is considered as an important imbalance.

^b^drug prescription for at least 3 consecutive months.

NTD, New Taiwan Dollar; CKD, chronic kidney disease; COPD, chronic obstructive pulmonary disease; PAOD, peripheral arterial occlusive disease; HT, hormone therapy

### Association between menopausal symptoms and CHD

**Fig 2:**
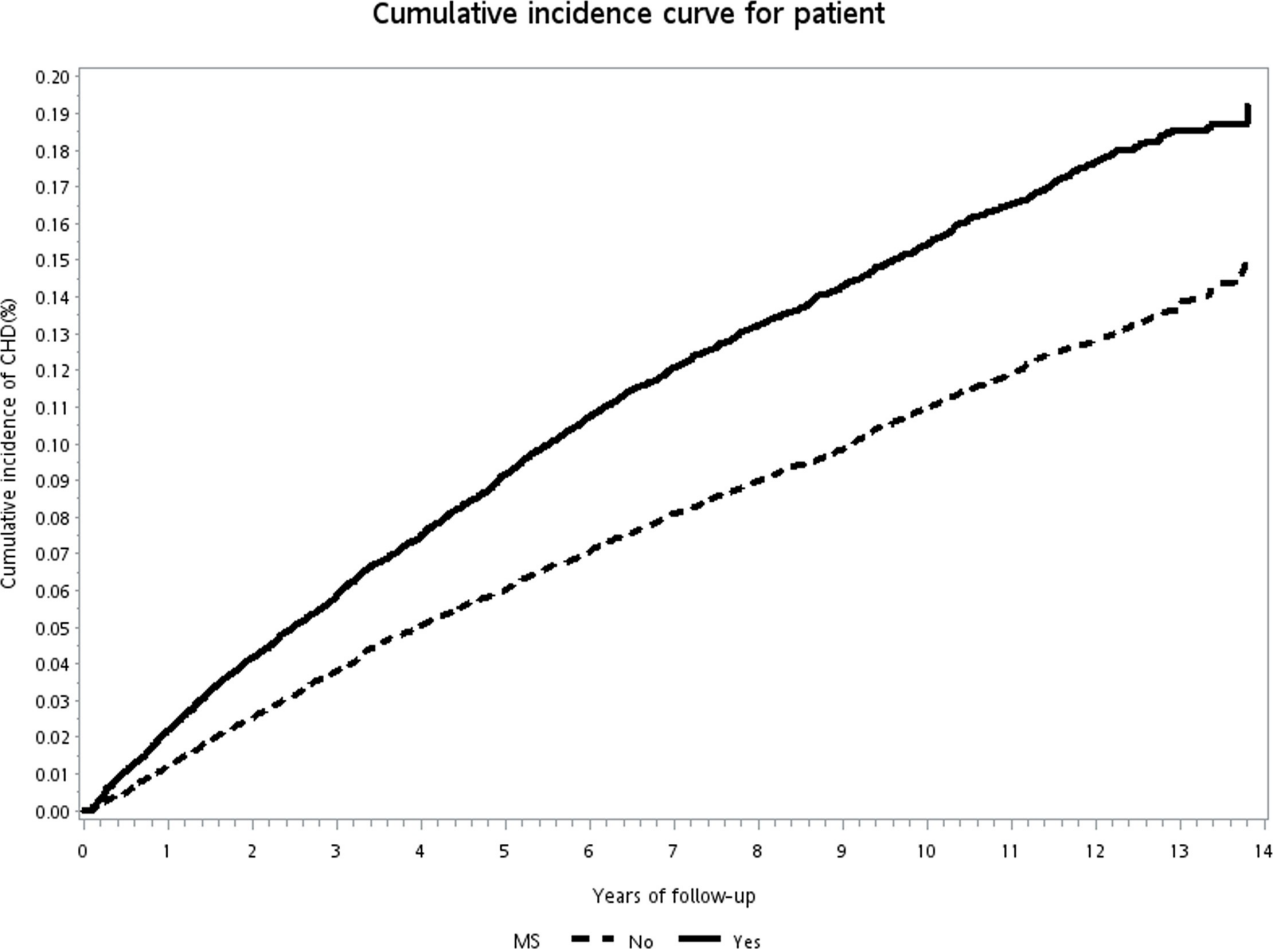
Cumulative incidence of CHD in symptomatic menopausal and control cohorts.

During the follow-up period, a total of 1429 (12.05%) and 1974 (17.18%) CHD events occurred in the control and symptomatic menopausal cohorts, respectively (**Table 2)**. Kaplan–Meier analysis revealed that the cumulative incidence of CHD was significantly higher in the symptomatic menopausal cohort than in the control cohort (log-rank test, *P* < 0.001) **(Fig 2**). Four models were employed to adjust the risk of CHD in the symptomatic menopausal cohort in comparison with that in the control cohort (**Table 2**). In Model 1, after propensity score matching, the risk of CHD was significantly higher in the patients with symptomatic menopause than in the controls (crude HR = 1.441, 95% confidence interval [CI] = 1.346–1.542, *P* < 0.0001). In Model 2, after propensity score adjustment, the incidence of CHD remained higher in the symptomatic menopausal cohort than in the control cohort (aHR = 1.456, 95% CI = 1.359–1.56, *P* < 0.0001). In Model 3, after adjustment for all the confounders listed in **Table 1**, the risk of CHD remained higher in the symptomatic menopausal cohort than in the control cohort (aHR = 1.428, 95% CI = 1.327–1.536, *P* < 0.0001). In Model 4, which was the full adjustment model, the increased risk of CHD in the symptomatic menopausal cohort remained significant (aHR = 1.338, 95% CI = 1.237–1.448, *P* < 0.0001). In Model 3, baseline characteristics, including age, sex, monthly income, CCI scores, comorbidity, and long-term medications, were used as covariates and were adjusted for in multivariate Cox analysis. Because comorbidities and prescribed medication use may not be present at baseline but may occur during follow-up, comorbidities and prescribed medication use were modeled using time-dependent covariates for the survival analyses in Model 4.

**Table 2:** Incidence and risk of CHD in symptomatic menopausal and control cohorts.

CHD	Event	PY^†^	incidence^‡^	Model 1		Model 2		Model 3		Model 4	
				HR (95% CI)	P-value^‡^	Adj.HR (95% CI)	P-value^‡^	Adj.HR (95% CI)	P-value^‡^	Adj.HR (95% CI)	P-value^‡^
Control cohort	1429	118573.15	12.05 (11.43,12.68)	reference		reference		reference		reference	
MS cohort	1974	114899.24	17.18 (16.42,17.94)	1.441(1.346,1.542)	< .0001	1.456(1.359,1.56)	< .0001	1.428(1.327,1.536)	< .0001	1.338(1.237,1.448)	< .0001

Model 1: Propensity score matched.

Model 2: Adjusted for propensity scores.

Model 3: Adjusted for all variables listed in Table 1. Table 1 variables including age, CCI score, monthly income, comorbidities, long-term use of medications.

Model 4: Adjusted for all variables listed in Table 1, and comorbidities and medication use were time-dependent covariates.

^†^PY: person-years; ^‡^per 1000 person-years; HR: hazard ratio; Adj. HR: adjusted hazard ratio; CI: confidence interval.

^‡^ All variables incorporated as competing risks of death.

CHD: coronary heart disease; MS: menopausal symptoms.

### Subgroup analyses of the risk of CHD between symptomatic menopausal and control cohorts

Subgroup analyses were performed to determine the association between symptomatic menopause and the risk of CHD for the age groups of <50 years and ≥50 years, subgroups without and with comorbidity, subgroups without and with HT, and subgroups of the different monthly income categories in both cohorts. The results revealed a statistically significant interaction effect between comorbidity and symptomatic menopause (*P* = 0.0217, **Table 3**). This finding indicates that symptomatic menopause had a differential effect on comorbidity. The reference group was the nonsymptomatic menopausal cohort. In this study, interaction effects were only assessed between symptomatic menopause and categorical variables. By employing various statistical models for controlling variables, we found that the symptomatic menopausal cohort had a significantly higher risk of CHD in all subgroups, except for the HT subgroup (**Table 3**).

**Table 3:** Subgroup analyses of the risk of CHD in symptomatic menopausal and control cohorts.

Subgroup	Subjects without MS		Subjects with MS		Compared to control group				
	N	Event	n	Event	aHR (95% CI)§	P-value	aHR (95% CI)^‡^	P-value	P_interaction_
Age, years									0.7178
<50	6274	366	6094	511	1.491(1.295,1.718)	< .0001	1.379(1.186,1.604)	< .0001	
≥50	8066	1063	8246	1463	1.398(1.284,1.522)	< .0001	1.326(1.209,1.453)	< .0001	
Comorbidity									0.0217
0	10965	779	10866	1157	1.548(1.406,1.704)	< .0001	1.435(1.291,1.594)	< .0001	
≥1	3375	650	3474	817	1.284(1.151,1.433)	< .0001	1.228(1.092,1.382)	0.0006	
Hormone therapy									0.5960
No	14057	1385	11374	1461	1.442(1.339,1.554)	< .0001	1.352(1.246,1.466)	< .0001	
Yes	283	44	2966	513	1.214(0.884,1.667)	0.2302	1.206(0.882,1.648)	0.2408	
Monthly income, NTD									0.5018
<15840	6200	765	6327	1073	1.421(1.286,1.57)	< .0001	1.356(1.218,1.51)	< .0001	
15840–25000	5254	520	5077	658	1.368(1.209,1.548)	< .0001	1.266(1.106,1.449)	0.0006	
≥25000	2886	144	2936	243	1.687(1.36,2.093)	< .0001	1.518(1.21,1.906)	0.0003	

^§^Model was adjusted for all variables listed in Table 1.

^‡^Model was adjusted for all variables listed in Table 1, and comorbidities and medication use were time-dependent covariates.

All analyses incorporated with regard to death as competing risks.

CHD: coronary heart disease; MS: menopausal symptoms

### Cumulative incidence of CHD stratified by symptomatic menopause and HT

We stratified the patients into four groups according to whether they had symptomatic menopause and whether they received HT. According to Kaplan–Meier survival analysis, the cumulative incidence of CHD was the lowest in the patients without symptomatic menopause who did not receive HT (log-rank test, *P* < 0.001; **S1 Fig)**. This group of patients was defined as the reference group, as presented in **S1 Fig** (MS = 0, HT = 0). The four models in **Table 2** were used to adjust the risk of CHD in the other three groups in comparison with that in the reference group (**S3 Table**). In Model 1, after propensity score matching, the risk of CHD was significantly higher in the other three groups than in the reference group. After adjustment in different statistical models (Models 2, 3, and 4), the incidence of CHD remained higher in the symptomatic menopausal patients with or without HT than it did in the reference group.

### HT and CHD risk

All patients who received HT had an increased risk of CHD (crude HR = 1.409, 95% CI = 1.305–1.52, *P* < .0001); however, these results were nonsignificant after adjustment for the variables listed in **Table 1**, including age, sex, monthly income, CCI scores, comorbidity, and long-term medications (aHR = 1.076, 95% CI = 0.985–1.176, *P =* 0.1031; **Table 4**). According to our analysis stratified by HT, aHRs for CHD were 1.082 (95% CI = 0.986–1.187, *P* = 0.0953) and 1.306 (95% CI = 0.998–1.71, *P* = 0.0517) in the symptomatic menopausal and control cohorts, respectively.

**Table 4:** HT and the Risk of CHD.

	All patient				Symptomatic menopausal cohort patient		Control cohort patient	
	cHR (95% CI)^§^	P-value	aHR (95% CI)^‡^	P-value	aHR (95% CI)^‡^	P-value	aHR (95% CI)^‡^	P-value
CHD								
Non-user	1(reference)		1(reference)		1(reference)		1(reference)	
HT user^※^	1.409(1.305,1.52)	< .0001	1.076(0.985,1.176)	0.1031	1.082(0.986,1.187)	0.0953	1.306(0.998,1.71)	0.0517

^§^Model was adjusted for HT.

^‡^Model was adjusted for HT and all variables listed in Table 1.

^※^HT was used as a time-dependent covariate for eliminating the effects of immortal time bias.

All analyses incorporated with regard to death as competing risks.

HT: hormone therapy

## Discussion

### CHD and menopausal symptoms

The main finding of our study is the association between menopausal symptoms and the risk of CHD. Our findings reveal that the incidence of CHD was higher in the symptomatic menopausal cohort than in the control cohort (17.18 vs. 12.05 per 1000 person-years). After adjustment for possible confounders in different models, the symptomatic menopausal cohort had a 1.338–1.441-fold higher risk of CHD than the control cohort did (**Table 2, Fig 2**). A recent systematic review and meta-analysis demonstrated that menopausal symptoms are associated with an increased risk of CVD, which is primarily explained by the associated CVD risk factors [16]. Of the 10 studies included in the review, only 1 (WHI-OS18) examined the association between vasomotor symptoms and the risk of CVD. The present study employed the propensity score matching method and CCI scores to balance baseline comorbidities and associated CVD risk factors between the two study cohorts. The strength of this study is the availability of information on possible confounders in the two cohorts. Thus, the association between menopausal symptoms and the risk of CHD could easily be determined through adjustment for these confounders.

A longstanding belief is that women exhibit a higher prevalence of metabolic syndrome (MetS) and a higher risk of CVD after menopause [17,18]. However, a recent study indicated that the severity of MetS and its five risk factors for heart disease may increase more rapidly before menopause rather than after menopause [19]. These findings may have clinical implications regarding the timing of cardiovascular risk development relative to menopause. When women undergo menopause, they may exhibit vasomotor or other menopausal symptoms before regular menses termination [20], and they may also be at an increased risk of CHD, according to our study findings. Therefore, to reduce the risk of CVD, health care providers should ensure appropriate diet and exercise and an overall healthy lifestyle among these women.

### HT and CHD risk

In the present study, the mean duration of HT was 3.3 ± 3.4 years in the symptomatic menopausal cohort and 1.3 ± 2.3 years in the control cohort. Overall, the mean duration of HT was 3.0 ± 3.3 years (**Table 1**). Our findings reveal that the patients undergoing HT had a nonsignificantly higher risk of CHD, regardless of the presence or absence of menopausal symptoms (**Table 4**). The strength of our study is that the data of the patients who received HT were censored 6 months after HT discontinuation to avoid neutralization of the results from past users. A randomized trial revealed that young healthy women who receive HT within 10 years of menopause onset do not have an increased risk of CHD [21]. The Kronos Early Estrogen Prevention Study (KEEPS) excluded participants with measurable coronary levels of calcium, which did not change over a 4-year period [22]. In our study, the mean age and HT duration in the symptomatic menopausal cohort were 52 and 3.3 years, respectively. We excluded patients with CHD and heart failure at baseline, and HT had a nonsignificant effect on the risk of CHD. Our results are similar to those of KEEPS, which revealed no differences in the risk of CHD between HT users and non-HT users. We postulate that the relative health of the study patients and the insufficient follow-up period prevented the observation of divergent effects.

## Study limitations

The present study has several limitations. First, the NHIRD does not include detailed laboratory data or detailed information on all socioeconomic parameters, smoking habits, body mass index (BMI), and blood pressure. The lack of adjustment for BMI, blood pressure, and smoking is a clear limitation that increased the risk of residual confounding. Second, we used the monthly income calculated from the NHI premium as a proxy for individual SES, but we could not determine household disposable income due to the lack of information on socioeconomic variables in the NHIRD. Third, symptomatic menopausal transition includes various somatic and psychiatric symptoms, but the NHIRD does not identify the symptoms on which gynecologists based their diagnoses. Furthermore, we could not assess the severity of menopausal symptoms because the data did not contain related clinical parameters. Therefore, we were unable to evaluate the relationship between SES and the severity of menopausal symptoms. Further clinical study is required to investigate the association between different menopausal symptoms and CVD development. Fourth, this study applied a retrospective design; therefore, additional prospective studies are necessary to confirm our findings. Finally, because the majority of Taiwan’s population is from a Chinese ethnic background, the findings of this study may not be generalizable to populations of different ethnicities.

## Conclusion

This population-based retrospective cohort study revealed that menopausal symptoms are a significant and independent risk factor for CHD among middle-aged women in Taiwan. Moreover, our findings indicate that HT has a nonsignificant effect on the risk of CHD.

## Supporting information

S1 Fig Cumulative incidence of CHD stratified by symptomatic menopause and HT. (TIF)

S1 Table The propensity-score model results of probability of menopausal symptoms. (DOCX)

S2 Table Validation of the symptomatic menopausal diagnosis by corresponding medications. (DOCX)

S3 Table Cumulative incidence of CHD stratified by symptomatic menopause and HT. (DOCX)

S1 Editing Certificate (PDF)

## Abbreviations

aHR: adjusted hazard ratio
CCI: Charlson comorbidity index
CHD: coronary heart disease
CI: confidence interva
CVD: cardiovascular disease
GYN: gynecology
HT: hormone therapy
HR: hazard ratio
ICD-9-CM: International Classification of Diseases, Ninth Revision, Clinical Modification
MS: menopausal symptoms
NHIRD: National Health Insurance Research Database
SD: standard deviation
SES: socioeconomic status
StD: standardized difference

## References

[1] Kannel WB, Hjortland MC, McNamara PM, Gordon T (1976) Menopause and risk of cardiovascular disease: the Framingham study. Annals of internal medicine 85: 447–452. 970770 10.7326/0003-4819-85-4-447

[2] Atsma F, Bartelink M-LE, Grobbee DE, van der Schouw YT (2006) Postmenopausal status and early menopause as independent risk factors for cardiovascular disease: a meta-analysis. Menopause 13: 265–279. 10.1097/01.gme.0000218683.97338.ea 16645540

[3] Reed S, Lampe J, Qu C, Gundersen G, Fuller S, Copeland W, et al (2013) Self-reported menopausal symptoms in a racially diverse population and soy food consumption. Maturitas 75: 152–158. 10.1016/j.maturitas.2013.03.003 23562010

[4] Gold EB, Colvin A, Avis N, Bromberger J, Greendale GA, Powell L, et al (2006) Longitudinal analysis of the association between vasomotor symptoms and race/ethnicity across the menopausal transition: study of women’s health across the nation. American journal of public health 96: 1226–1235. 10.2105/AJPH.2005.066936 16735636 PMC1483882

[5] Herber‐Gast G, Brown W, Mishra G (2015) Hot flushes and night sweats are associated with coronary heart disease risk in midlife: a longitudinal study. BJOG: An International Journal of Obstetrics & Gynaecology 122: 1560–1567. 25377022 10.1111/1471-0528.13163

[6] Gast G-CM, Pop VJ, Samsioe GN, Grobbee DE, Nilsson PM, Keyzer JJ, et al (2011) Vasomotor menopausal symptoms are associated with increased risk of coronary heart disease. Menopause 18: 146–151. 10.1097/gme.0b013e3181f464fb 21127438

[7] Szmuilowicz ED, Manson JE, Rossouw JE, Howard BV, Margolis KL, Greep NC, et al (2011) Vasomotor symptoms and cardiovascular events in postmenopausal women. Menopause (New York, NY) 18: 603. 10.1097/gme.0b013e3182014849 PMC3123435 21358352

[8] Matthews KA, Crawford SL, Chae CU, Everson-Rose SA, Sowers MF, Sternfeld B, et al (2009) Are changes in cardiovascular disease risk factors in midlife women due to chronological aging or to the menopausal transition? Journal of the American College of Cardiology 54: 2366–2373. 10.1016/j.jacc.2009.10.009 20082925 PMC2856606

[9] Chen M-H, Su T-P, Li C-T, Chang W-H, Chen T-J, Bai Y-M. (2013) Symptomatic menopausal transition increases the risk of new-onset depressive disorder in later life: a nationwide prospective cohort study in Taiwan. PloS one 8: e59899 10.1371/journal.pone.0059899 23544108 PMC3609738

[10] Lien H-M, Chou S-Y, Liu J-T (2008) Hospital ownership and performance: evidence from stroke and cardiac treatment in Taiwan. Journal of health economics 27: 1208–1223. 10.1016/j.jhealeco.2008.03.002 18486978

[11] Cheng C-L, Chien H-C, Lee C-H, Lin S-J, Yang Y-HK (2015) Validity of in-hospital mortality data among patients with acute myocardial infarction or stroke in National Health Insurance Research Database in Taiwan. International journal of cardiology 201: 96–101. 10.1016/j.ijcard.2015.07.075 26292275

[12] Wu C-Y, Chen Y-J, Ho HJ, Hsu Y-C, Kuo KN, Wu M-S, et al (2012) Association between nucleoside analogues and risk of hepatitis B virus–related hepatocellular carcinoma recurrence following liver resection. Jama 308: 1906–1913. 23162861 10.1001/2012.jama.11975

[13] Charlson ME, Pompei P, Ales KL, MacKenzie CR (1987) A new method of classifying prognostic comorbidity in longitudinal studies: development and validation. Journal of chronic diseases 40: 373–383. 3558716 10.1016/0021-9681(87)90171-8

[14] de Groot V, Beckerman H, Lankhorst GJ, Bouter LM (2003) How to measure comorbidity: a critical review of available methods. Journal of clinical epidemiology 56: 221–229. 12725876 10.1016/s0895-4356(02)00585-1

[15] Fine JP, Gray RJ (1999) A proportional hazards model for the subdistribution of a competing risk. Journal of the American statistical association 94: 496–509.

[16] Muka T, Oliver-Williams C, Colpani V, Kunutsor S, Chowdhury S, Chowdhury R, et al (2016) Association of vasomotor and other menopausal symptoms with risk of cardiovascular disease: a systematic review and meta-analysis. PloS one 11: e0157417 10.1371/journal.pone.0157417 27315068 PMC4912069

[17] Janssen I, Powell LH, Crawford S, Lasley B, Sutton-Tyrrell K (2008) Menopause and the metabolic syndrome: the Study of Women's Health Across the Nation. Archives of internal medicine 168: 1568–1575. 10.1001/archinte.168.14.1568 18663170 PMC2894539

[18] Colditz GA, Willett WC, Stampfer MJ, Rosner B, Speizer FE, Hennekens CH. (1987) Menopause and the risk of coronary heart disease in women. New England Journal of Medicine 316: 1105–1110. 10.1056/NEJM198704303161801 3574358

[19] Gurka MJ, Vishnu A, Santen RJ, DeBoer MD (2016) Progression of metabolic syndrome severity during the menopausal transition. Journal of the American Heart Association 5: e003609 10.1161/JAHA.116.003609 27487829 PMC5015287

[20] Reed SD, Lampe JW, Qu C, Copeland WK, Gundersen G, Fuller S, et al (2014) Premenopausal vasomotor symptoms in an ethnically diverse population. Menopause 21: 153–158. 10.1097/GME.0b013e3182952228 23760434

[21] Lobo RA (2017) Hormone-replacement therapy: current thinking. Nature Reviews Endocrinology 13: 220–231. 10.1038/nrendo.2016.164 27716751

[22] Harman SM, Black DM, Naftolin F, Brinton EA, Budoff MJ, Cedars MI, et al (2014) Arterial Imaging Outcomes and Cardiovascular Risk Factors in Recently Menopausal WomenA Randomized TrialCardiovascular Disease and Menopausal Hormone Therapy. Annals of internal medicine 161: 249–260. 10.7326/M14-0353 25069991

