# Supplementary figures and images for: Menopausal symptoms and risk of coronary heart disease in middle-aged women: A nationwide population-based cohort study
Source: PLoS One. 2018 Oct 18;13(10):e0206036. doi: 10.1371/journal.pone.0206036 (PMC6193730; doi:10.1371/journal.pone.0206036)

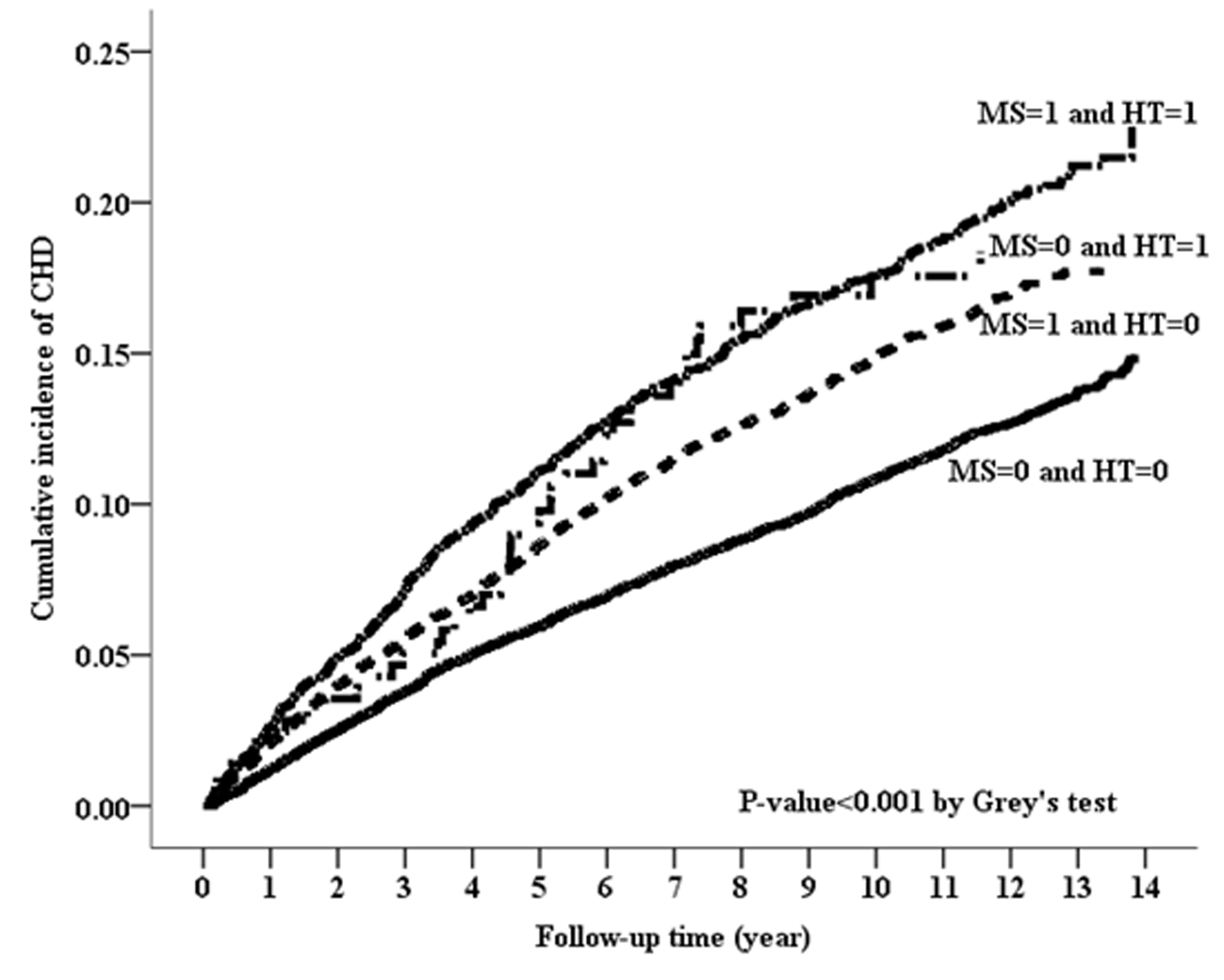

Supplement: S1 Fig — (TIF) [file pone.0206036.s001.tif]
