# Supplementary material for: Menopausal symptoms and risk of coronary heart disease in middle-aged women: A nationwide population-based cohort study
Source: PLoS One. 2018 Oct 18;13(10):e0206036. doi: 10.1371/journal.pone.0206036 (PMC6193730; doi:10.1371/journal.pone.0206036)
Supplement: S1 Table — (DOCX) [file pone.0206036.s002.docx]

**S1 Table. The propensity-score model results of probability of menopausal symptoms**

|  | **Estimate** | **OR** | **95% CI for OR** | | **p-value** |
| --- | --- | --- | --- | --- | --- |
| **Monthly income (per NTD 10000)** | **0.066** | **1.068** | **1.057** | **1.080** | **<.0001** |
| **Hypertension** | **-0.204** | **0.816** | **0.774** | **0.859** | **<.0001** |
| **Hyperlipidemia** | **0.499** | **1.648** | **1.548** | **1.754** | **<.0001** |
| **Diabetes mellitus** | **-0.149** | **0.862** | **0.782** | **0.949** | **0.003** |
| **Obesity** | **-0.194** | **0.824** | **0.646** | **1.051** | **0.119** |
| **CKD** | **0.039** | **1.040** | **0.919** | **1.177** | **0.537** |
| **Stroke** | **-0.545** | **0.580** | **0.516** | **0.651** | **<.0001** |
| **COPD** | **0.392** | **1.481** | **1.387** | **1.580** | **<.0001** |
| **PAOD** | **0.247** | **1.280** | **1.002** | **1.634** | **0.048** |
| **Dysarrhythmia** | **0.404** | **1.497** | **1.324** | **1.693** | **<.0001** |
| **Diabetic drugs** | **-0.434** | **0.648** | **0.572** | **0.734** | **<.0001** |
| **Statin** | **-0.442** | **0.643** | **0.576** | **0.718** | **<.0001** |
| **Anti-hypertension** | **-0.227** | **0.797** | **0.748** | **0.850** | **<.0001** |
