# Supplementary material for: Menopausal symptoms and risk of coronary heart disease in middle-aged women: A nationwide population-based cohort study
Source: PLoS One. 2018 Oct 18;13(10):e0206036. doi: 10.1371/journal.pone.0206036 (PMC6193730; doi:10.1371/journal.pone.0206036)
Supplement: S2 Table — (DOCX) [file pone.0206036.s003.docx]

**S2 Table. Validation of the symptomatic menopausal diagnosis by** **corresponding medications**

|  | menopausal drug=0 | menopausal drug=1 |
| --- | --- | --- |
| menopausal symptoms=0 | 8515 (59.38%) | 5825 (40.62%) |
| menopausal symptoms=1 | 1189(8.29%) | 13151(91.71%) |

Menopausal symptoms=0: indicated subjects without symptomatic menopausal diagnostic code (ICD-9-CM code: 627.2)

Menopausal symptoms=1: indicated subjects with symptomatic menopausal diagnostic code

Menopausal drug=0: indicated subjects without receiving corresponding medications after diagnostic code allocation

Menopausal drug=1 indicated subjects receiving corresponding medications after diagnostic code allocation
