# Supplementary material for: Menopausal symptoms and risk of coronary heart disease in middle-aged women: A nationwide population-based cohort study
Source: PLoS One. 2018 Oct 18;13(10):e0206036. doi: 10.1371/journal.pone.0206036 (PMC6193730; doi:10.1371/journal.pone.0206036)
Supplement: S3 Table — (DOCX) [file pone.0206036.s004.docx]

**S3 Table. Cumulative Incidence of CHD Stratified by Symptomatic Menopause and HT**

| **CHD** | Model 1 | | Model 2 | | Model 3 | | Model 4 | |
| --- | --- | --- | --- | --- | --- | --- | --- | --- |
|  | HR  (95% CI) | P-value^‡^ | Adj.HR  (95% CI) | P-value^‡^ | Adj.HR (95% CI) | P-value^‡^ | Adj.HR  (95% CI) | P-value^‡^ |
| Control cohort | reference | | reference | | reference | | reference | |
| MS=0 and HT=1 | 1.55(1.14,2.09) | 0.005 | 1.28(0.95,1.74) | 0.118 | 1.26(0.93,1.72) | 0.134 | 1.09(0.78,1.51) | 0.6275 |
| MS=1 and HT=0 | 1.39(1.29,1.49) | <0.001 | 1.44(1.34,1.55) | <0.001 | 1.44(1.34,1.55) | <0.001 | 1.35(1.24,1.46) | <0.001 |
| MS=1 and HT=1 | 1.70(1.53,1.88) | <0.001 | 1.55(1.4,1.72) | <0.001 | 1.53(1.38,1.70) | <0.001 | 1.34(1.14,1.57) | <0.001 |

Model 1: Propensity score matched.

Model 2: Adjusted for propensity scores.

Model 3: Adjusted for all variables listed in Table 1.

Model 4: Adjusted for all variables listed in Table 1, and comorbidities and medication use were time-dependent covariates.

HR: hazard ratio; Adj. HR: adjusted hazard ratio; CI: confidence interval.

^‡^ All variables incorporated as competing risks of death.

CHD: coronary heart disease; MS: menopausal symptoms; HT: hormone therapy
