# Supplementary material for: Menopausal symptoms and risk of coronary heart disease in middle-aged women: A nationwide population-based cohort study
Source: PLoS One. 2018 Oct 18;13(10):e0206036. doi: 10.1371/journal.pone.0206036 (PMC6193730; doi:10.1371/journal.pone.0206036)
Supplement: S1 Editing Certificate — (PDF) [file pone.0206036.s005.pdf]

Wallace Academic Editing

English Editing Certificate

This certifies that the paper **Menopausal symptoms and risk of coronary heart disease in middle-aged women: A nationwide population-based cohort study** has been edited by Katie Fonseca on September 29, 2018 and is considered to be improved in grammar, punctuation, spelling, verb usage, sentence structure, conciseness, general readability, writing style, and native English usage to the best of the editor's ability.

KF

Best regards,
Wallace Academic Editing

Phone No.: +886-2-2555-5830
Website: http://www.editing.tw
Email: editing@editing.tw
Address: 3F., No.180, Chang'an W. Rd., Datong Dist., Taipei City
